# Jamaican Mothers’ Perceptions of Children’s Strategies for Resisting Parental Rules and Requests

**DOI:** 10.3389/fpsyg.2018.01786

**Published:** 2018-09-21

**Authors:** Taniesha Burke, Leon Kuczynski

**Affiliations:** ^1^Department of Psychology, Universität Konstanz, Konstanz, Germany; ^2^Family Relations and Applied Nutrition, University of Guelph, Guelph, ON, Canada

**Keywords:** Jamaica, resistance strategies, non-compliance, child agency, parenting

## Abstract

Research on Jamaican socialization of children has primarily focused on parental discipline practices. Little is known about children’s responses to parental attempts to control their behavior. The present study investigated mothers’ perceptions of children’s strategies for resisting their rules and requests. Thirty mothers living in Kingston and St. Andrew, Jamaica, participated in a 1- to 1.5-h semi-structured, open-ended interview regarding their 8- to 12-year-old children. Mothers reported that their children’s resistance strategies included assertive refusal, arguing, ignoring/avoiding, attitude, and negotiation. Most mothers disapproved of their children’s actions and responded with power-assertive strategies such as physical punishment, psychological control, forced compliance, and threats. Few mothers responded with autonomy support strategies including accommodation and reasoning. The findings provided insight into the ways Jamaican children use their agency to protect their autonomy despite their mothers’ greater power, and the relational nature of children’s influence on their mothers’ behaviors and reactions. More research is needed to expand our knowledge of child agency in Afro-Caribbean families and the various ways that parents may support their growing autonomy that is socially constructive.

## Introduction

Research on Jamaican parent–child relationships is relatively new and encompasses questions concerning socialization processes (Chevannes, 2001; Leo-Rhynie and Brown, 2013), family structures (Leo-Rhynie, 1997), and parental control (Ricketts and Anderson, 2009; Smith et al., 2011). Theoretically, research on children’s socialization in the Jamaican family context has predominantly taken a traditional unilateral or parent-centered approach with an exclusive emphasis on how parents influence children through their discipline practices (Kuczynski, 2003). For example, although there have been some reports of authoritative parenting styles (Lipps et al., 2012), most studies suggest that Jamaican parents have been found to have an authoritarian parenting style, characterized by values that favor strict obedience from children and harsh, punitive discipline (UNICEF, 2010; Burke and Sutherland, 2014). The authoritarian style has been attributed to historical influences including slavery, colonialism, and conservative Christian religion (Barrow, 2001; Bush, 2010; Jemmott, 2015).

Research has documented that Jamaican parents frequently use corporal punishment, shaming, rejection, and humiliation to discipline and control their children (Ricketts and Anderson, 2008; UNICEF, 2010; Smith et al., 2011; Smith and Moore, 2013). However, there is evidence that parental values and disciplinary responses may vary by social class. Parents from lower socioeconomic backgrounds placed greater value on obedience, and manners as these characteristics are deemed necessary for social mobility (Roopnarine, 2006; Anderson, 2007). In contrast, middle class families supported their children’s autonomy by encouraging self-direction, assertiveness, dialogue, and independence (Anderson, 2007; Brown and Johnson, 2008). Older, affluent parents with a higher level of education were found to use less punitive approaches that included the removal of privileges and reasoning (Ricketts and Anderson, 2009), while others have recognized the benefits of child-centered responses that consider children’s needs and perspectives (Burke and Sutherland, 2014).

A drawback of the existing research is that the unilateral socialization theory framing of research questions has resulted in a narrow focus on parental discipline and has neglected the role of children as active agents, and sources of influence in parent–child interactions (Maccoby, 2000; Kuczynski, 2003; Grusec and Davidov, 2010). Instead, in most studies, Jamaican parents have been treated as unresponsive in their application of disciplinary tactics and Jamaican children as passive recipients of parental practices, with little consideration for the dynamic interpersonal context in which parent–child interactions occur.

Although not much evident in Jamaican research, or, more generally, in cross-cultural research on families, theoretical approaches to socialization since the 1980s have increasingly recognized that children are agents who contribute to socialization processes in parent–child relationships. Most contemporary theories of socialization place child agency and child influence within larger transactional and relational perspectives, which view causal processes in the family as inherently bidirectional such that both parents and children are agents engaged in mutual influence over time (Maccoby and Martin, 1983; Kuczynski, 2003; Sameroff, 2009). As agents in the family context, children construct their own meanings from parental demands and messages, and resist demands that they evaluate as unfair, wrong, or outside of parents’ jurisdiction (Smetana and Jambon, 2018). Moreover, children are strategic in achieving goals that may differ from those of their parents, and they strive to resist attempts to block their goals or their self-constructed understanding of experiences (Kuczynski and De Mol, 2015).

The perspective of child agency offers new interpretations of basic phenomena that have been traditionally understood using unilateral socialization perspectives. The most important example is the phenomenon of children’s non-conformity to parental requests and rules. In the traditional unilateral perspective, children’s refusal or attempt to evade a parental rule or request is conceptualized as *non-compliance* (Patterson, 1982; Baumrind, 2012). The term non-compliance reflects a parent-centered perspective and is defined as the failure to comply immediately and exactly to the parents’ requests or rules. The standard of strict obedience to authority is also a common expectation for appropriate parent–child relationships in collectivist cultures (Trommsdorff and Kornadt, 2003) and is associated with traditional authoritarian parenting styles (Kuczynski and Hildebrandt, 1997). In family interventions based on this perspective, parents are trained to view non-compliance as a deviant or problematic behavior that requires suppression (McMahon and Forehand, 2003).

The alternative perspective in several disciplines conceptualizes children’s non-conformity as *resistance.* In contrast to the construct of non-compliance, resistance connotes a focus on children’s non-conformity as an agentic and potentially constructive expression of children’s autonomy in the parent–child relationship (Wenar, 1982; Kuczynski et al., 1987; Crockenberg and Litman, 1990). Children’s resistance, in this view, reflects their developing motives to protect their freedom of thought and action in response to their parents’ attempts to control them. Thus, childhood resistance is conceptually related to adulthood concepts such as reactance to control (Brehm, 1981), self-determination (Deci and Ryan, 2000), and resistance to oppression (Goffman, 1961; Scott, 1990; Wade, 1997).

Considered in the context of an interpersonal relationship, young children’s expressions of resistance have been considered at different levels of analyses. Micro-level analyses of parent–child conversations suggest that power, authority, and the right for self-determined action are negotiated in a subtle, barely perceptible way as very young children deflect, evade, and challenge parental requests and claims to authority during moment to moment interchanges (Kent, 2012; Stevanovic, 2018). At a more molar level, analyses of less frequent parent–child conflict occasioned by children’s non-compliant responses to parental demands, standing rules, and prohibitions have been regarded as social strategies whereby children attempt to influence parents to drop or modify their demands (Kuczynski et al., 1987; Kuczynski and Kochanska, 1990).

In a program of research using North American samples, Kuczynski et al. (1987) and Kuczynski and Kochanska (1990) have mapped the development of children’s repertoires resistance strategies between the ages of 18 months and 18 years. Observational research with toddler and preschool children identified strategies including, ignoring, simple refusal, negotiation, explanation, and direct defiance (Kuczynski et al., 1987; Kuczynski and Kochanska, 1990). Developmental changes included skillful strategies such as negotiation increasing with age, and unassertive strategies for expressing autonomy such as ignoring decreasing between 18 months and 5 years. During middle childhood (Kuczynski et al., in press) and adolescence (Parkin and Kuczynski, 2012) similar categories of strategies for overtly resisting parental demands have been identified, albeit at greater levels of skill and assertiveness. Also, the use of contextualized qualitative methodologies such as event diaries and critical incident reports have identified covert forms of resistance such as negative cognitive evaluations of parental messages despite behavioral compliance or acquiescence, as well as covert transgressions and evasions of parental demands. Taken together, a picture is emerging from middle class North American samples whereby children develop their repertoires for expressing agency through resistance to parents along dimensions of social skill in negotiation, assertiveness in challenging parental authority, and modes of covert expression.

Research on parental responses to children’s acts of resistance is less rich but suggest that middle class North American parents interpret children’s expressions of resistance as normative expressions of autonomy that they tolerate and channel for the sake of developing children’s social competence. In particular, strategies such as simple assertion, explanation, and negotiation are experienced by parents as acceptable and legitimate expressions of children’s autonomy (Goodnow, 1997; Morrissey and Gondoli, 2012; Kuczynski et al., 2013).

An objective of the present research is to explore how Jamaican mothers interpret and respond to children’s resistance to their standing rules and requests. Research on Jamaican families has not previously investigated socialization processes using the theoretical construct of child agency and strategic resistance. Beyond extending this agentic framework, cross-culturally, the Jamaican family context is of theoretical interest because the form by which resistance occurs may be influenced by cultural values and expectations (Bornstein, 1995; Trommsdorff and Kornadt, 2003; Bornstein and Cheah, 2006). For instance, individualistic cultures place priority on fostering independence, self-assertion, a sense of autonomy, individual freedom and the maximization of individual goals. In contrast, collectivistic cultures place a higher value on cooperation and mutual respect. Relationships in these cultures are based on hierarchical power relations where the young must comply with the elders’ wishes (Tamis-LeMonda et al., 2008). The differences in behavioral expectations between individualistic and collectivistic cultures have implications on the strategies children use to resist restrictions on their agency.

Jamaica is a considered collectivistic country with strong evidence of hierarchical power relations, authoritarian relationships, and harsh discipline practices for non-conformity (Hofstede, 2018). The strong emphasis on obedience and harsh parental power suggest that Jamaican parents have the intention to suppress expressions of resistance in their children. Although, there is no research on whether or how children resist, there is research that suggests that Jamaican children do not passively accept the strict expectations of compliance or the harsh parenting that they receive. Some researchers have found that Jamaican children disapproved of their parents’ use of punitive parental control strategies and were perplexed by their parents’ justification that such practices are an expression of their love (Bailey et al., 1998; Brown and Johnson, 2008).

The research strategy of this study was to ask mothers to describe recent interactions in which their children challenged their authority or resisted complying with their requests. The qualitative methodology was designed to explore how mothers perceived and evaluated children’s resistance, and whether the kinds of strategies they identify in their children and their own reactions correspond to previously reported North American research. The middle childhood period is understudied in the Jamaican literature and; moreover, children in middle childhood experience greater independence away from home, have increased capacity to self-regulate (Maccoby, 1984), and evaluate their actions (Eccles, 1999), and enhanced cognitive skills (Collins et al., 2002).

Theoretically, social relational theory guided this research (Kuczynski and De Mol, 2015). The theory places child agency and bidirectional influence between parents and children in the distinctive context of the parent–child relationship. According to social relational theory parents and children are both considered human agents who are causally connected in a culturally embedded, interdependent, long-term, close relationship which influences how they experience and express their agency in social interactions (Kuczynski and De Mol, 2015). Culture is assumed to influence bidirectional dynamics because of varying norms for appropriate power relations and standards of communication between parents and children. Thus, although children in all cultures are agents, how they experience and express their agency is dependent on culturally embedded relationship contexts. In the present study, key features of the Jamaican parent–child relationship that have a direct bearing on children’s expression of agency through resistance and mothers’ responses to them are the collectivist culture, norms for hierarchical power relations requiring respect for elders, and norms for harsh punitive consequences for infractions.

## Materials and Methods

### Participants

Thirty mothers (*M*_age_ = 39.43 years, range 29–50 years; *SD* = 5.57 years) were recruited to participate in a semi-structured interview. On average, they had 2.5 children. **Table 1** contains a detailed description of participants’ marital status, education level, employment status, and living arrangements. Participants lived in the metropolitan area of Kingston and St. Andrew, Jamaica. Participants were classified into middle class (*n* = 17) and lower class (*n* = 13), which was determined with the aid of the Statistical Institute of Jamaica 2011 census maps of enumerated districts, and participants’ demographic information. The combined information determined the communities that were known to have a history of violence and low income. The marital status of the sample is consistent with earlier findings that have shown that 84% of Jamaican children were born out of wedlock and most children born in wedlock were from the middle class (National Family Planning Board, 2008).

**Table 1:** Description of sample characteristics.

Variables	Middle class	Lower class
	*n* = 17	*n* = 13
**Marital status**		
Married	14 (82%)	2 (15%)
Divorced	1 (6%)	0
Common-law	2 (12%)	5 (39%)
Single	0	6 (46%)
**Education level**		
University	16 (94%)	3 (23%)
Community college	1 (6%)	1 (8%)
HEART-NTA	0	2 (15%)
Other post-secondary	0	3 (23%)
Secondary	0	4 (31%)
**Employment status**		
Full-time	13 (77%)	6 (46%)
Part-time	3 (18%)	2 (15%)
Self-employed	1 (6%)	3 (23%)
Unemployed	0	2 (15%


*HEART-NTA means Human Employment and Resource Training – National Training Agency Certification. Other post-secondary includes accounting school, cosmetology school, and university continuing studies.*

Criteria for participation included living in the Kingston and St. Andrew metropolitan area, and being the biological mother of at least one child who was between the ages of 8 and 12 (*M*_age_ = 10.47 years, *SD* = 1.43). There were 16 girls and 14 boys who were the subjects of the interviews. The results are categorized by social class because previous findings have found that parents from both classes have different expectations of children’s autonomy that may influence the form and frequency of children’s expression of resistance (Anderson, 2007; Brown and Johnson, 2008).

Mothers were selected as participants for the study because matrilineal families are the hallmark of the Jamaican culture and the reverence given to women who have children, (Sargent and Harris, 1992; Bush, 2010). Furthermore, the Jamaica Survey of Living conditions found that 46% of all Jamaican households are headed by females, and 75% of those households do not have a male spouse present, which has resulted in roughly one-third of children not having father figure resident in the home (The Planning Institute of Jamaica, 2012). The demographic structure of Jamaican homes means that mothers are primarily the socializing agents compared to fathers and thus, enjoy a closer emotional connection with them. Based on our selection criteria and areas of focus a purposive sampling method was used to recruit participants.

### Procedure

Ethics review boards in Jamaica and Canada approved the research design. Participants were recruited with the assistance of community leaders, pastors, human resource managers in companies located in Kingston and St. Andrew, personal contacts, and participants. The local contacts received a flyer that contained details of the study’s purpose, requirements for participant inclusion, and compensation, which they distributed. Participants were also asked to connect the authors to other mothers in their network who met the inclusion requirements.

Participants’ consented before the commencement of the interviews. They were asked to participate in an open-ended 1 to 1.5-h semi-structured interview in their home or a mutually convenient location. The interviews were conducted by the first author using the critical incident technique, which is a procedure designed to collect narrative descriptions about people’s contextualized experiences in specified naturally occurring events (Butterfield et al., 2005). Participants were asked to recall in detail four recent incidents where their children resisted their rules and requests, four incidents where they responded to children’s distress and four incidences when they felt close to their children. This paper only includes the parental rules data. At the end of the interview, each participant received a USD $2 prepaid calling card.

In order to build rapport, the interviewer identified herself as a Jamaican native born and raised in Jamaica as well as a mother and related to participants based on their environmental contexts. For example, the interview was conducted in the Jamaican dialect, Patois, if it made the participants feel more comfortable. The decision to self-disclose was to foster dialogue that included trust, honesty, openness, and equality between the researcher and participants (Hayman et al., 2012). All interviews were audio-recorded and transcribed verbatim by native Jamaicans to maintain authenticity. The first author conducted the initial reading and coding of the transcripts with the aid of MAXQDA software.

### Theoretical Approach to Qualitative Method

Our theoretical approach to this qualitative study was interpretive phenomenology. The focus is to understand, describe and interpret mothers’ subjective lived experiences when interacting with their children, within various social, cultural, psychological and theoretical contexts (Holloway and Todres, 2003; Starks and Brown Trinidad, 2007). Procedurally, the analysis followed Braun and Clarke’s (2006) steps for theoretical thematic analysis. These consisted of familiarizing ourselves with the data, generating initial codes, developing and reviewing themes, defining and naming themes, and presenting the final themes.

Existing theories on parent–child relationships and the first author’s knowledge of the Jamaican culture were used as sensitizing concepts during the initial stages of analyses (Kuczynski and Daly, 2003). However, the researchers were also alerted to novel concepts that contradicted or not considered ideas in the research or theoretical literature. To ensure trustworthiness of the findings, we created audit trails that included documentation of daily logs and memos that recorded systematic categorization of data, theme development, analyses, and justification of final themes (Lincoln and Guba, 1985). Regular meetings with the second author ensured that the themes represented participants’ description of their experiences, increased the scope of interpretations and identified unique aspects of Jamaican culture that might have been missed by the first author because to her native familiarity with the culture.

## Results

The analyses addressed three overarching questions: (1) mothers’ perceptions of children’s resistance strategies; (2) mothers’ appraisals of children’s resistance to their requests; and (3) mothers’ responses to children’s resistance. Each section will present the themes and subthemes that were identified along with quotations to illustrate the themes. Quotations from participants are presented in the original Jamaican Patois to maintain the authenticity of the narratives. The tables report the number of respondents who mentioned a theme as an aid in interpreting the data. However, the numbers are not an indication of statistical significance because the sample size was too small for statistical analyses. The supporting quotes are identified by social class of parent (MC, middle class; LC, lower class) and the age and gender of the index child.

### Mothers’ Perceptions of Children’s Non-compliance and Resistance

All mothers reported recent incidents when their children resisted or did not comply with their expectations and identified their children’s strategies for resisting or avoiding complying with the parents’ wishes. The contexts of children’s resistance predominantly consisted of immediate requests and prohibitions as well as standing rules that represented expectations for behavior issued in the past. The specific topics of resistance pertained to expectations concerning safety, homework, and chores each reported by 50% of our sample of mothers, followed by etiquette and hygiene (43%); sibling conflict (27%), meal and bedtime routines (27%) and junk-food consumption (20%). The thematic analyses of mothers’ reports identified five distinct categories of resistance: *assertive refusal, arguing, ignoring/avoiding, displaying attitude, and negotiation* (**Table 2**).

**Table 2:** Themes in mothers’ perceptions of children’s non-compliance and resistance strategies.

	Middle	Lower	Total
	class	class	(*N* = 30)
	(*n* = 17)	(*n* = 13)	
Assertive refusal	71% (12)	62% (8)	67% (20)
Arguing	65% (11)	54% (7)	60% (18)
Ignoring/avoiding	65% (11)	69% (9)	67% (20)
Attitude	59% (10)	62% (8)	60% (18)
Negotiation	77% (13)	62% (8)	70% (21)


*Table includes percentage of mothers. Absolute values are in parentheses.*

#### Assertive Refusal

More than half the mothers from both social classes described their children as directly refusing in a simple assertive manner by saying ‘no’ without displays of negative affect or verbal coercion. For instance, one mother reported that her child refused to complete her chores by saying, ‘No, no I am not doing it’ (MC, 8-year-old daughter) in response to a request to complete a task.

#### Arguing

Arguing refers to mothers’ reports that children verbally confronted them with excuses, demands for explanations or angry verbal challenges. More middle class mothers reported that their children resisted through arguing compared to lower class mothers and may indicate that middle class mothers were more likely to provide leeway (**Table 2**). One of the major examples of arguing was their children’s use of what mothers referred to as ‘back-talking,’ which involved verbally challenging a statement made by mothers instead of complying without objections. “I was saying something to her and she answered back” (LC, 12-year-old daughter). Mothers also described children as intentionally arguing with them to avoid requests/demands such as chores and bedtime routines. One mother described her experience with her son in relation to his bedtime routine, “I have mentioned of him being argumentative. It isn’t even talkative. It is argumentative. He will just test everything” (MC, 9-year-old son).

#### Ignoring/Avoiding

In contrast to assertive refusals, mothers described instances of resistance where children did not directly confront mothers with their opposition. Instead, mothers reported that their children ignored their requests in an unassertive manner, pretended not to hear, or delayed completing chores with non-committal responses. For example, “You call S (the daughter), “S,” you cyaah hear she ansa. “S,” no ansa, and den yuh jus see she appear” (LC, 12-year-old daughter). Mothers reported children avoided request/demands relating to homework by inserting their priorities. “She is preparing for her GSAT (Grade Six Achievement Test), and so she had gotten a number of quizzes that she need to complete...It was a very, very busy and chaotic holiday. . . But she knew she had them to do and she kept trying to go around them” (MC, 12-year-old daughter).

#### Attitude

Mothers reported instances where children complied with their requests to not, for example, eat junk food, harass their siblings, and to complete their chores but conveyed their non-acceptance of mothers’ authority with non-verbal challenges, which mothers described as “attitude.” Attitude was considered as a subversive form of resistance because, although children complied, they displayed disapproving facial expressions, body language, gestures, sounds, and tone of voice. Most of the mothers’ descriptions of attitude concerned their daughters. One mother, in particular, described her daughter’s response when she told her that her she had to treat her brother well, “She tends to puff when she does not get her own way” (MC, 8-year-old daughter). Another mother shared a similar reaction when she instructed her daughter to complete her chores, “I talk to her and she a makeup har face …and she a huff and a puff” (LC, 10-year-old daughter).

Hissing of the teeth, which is an expression of attitude that is characteristically found among the African diaspora in the Caribbean, was also reported by only lower class mothers as a form of resistance. Hissing of teeth is also referred to as ‘suck teeth’ or ‘kiss-teeth.’ This is the “gesture of drawing air through the teeth and into the mouth to produce a loud sucking sound” (Rickford and Rickford, 1976, pp. 302–303). For example, one mother shared her son’s reaction to her request to not leave the confines of their home to play with friends, “A pure hiss teeth ting him do sometime” (LC, 12-year-old son).

#### Negotiation

Most mothers reported that their children used negotiation strategies whereby children verbally attempted to accommodate mothers’ wishes or persuade mothers to modify the nature of their demands/requests. Some examples of negotiation were perceived as cooperative. Mothers reported that children conveyed that they were willing to do what was requested but asked mothers to justify their requirements. Mothers said that children often attempted to negotiated changes to the time frame in which they were expected to comply with general family routines. “Sometime she needs to go to the hairdresser and she doesn’t want to go and she might tell you tomorrow and then tomorrow it doesn’t work out” (MC, 9-year-old daughter). Mothers also described children as providing explanations for their resisting or requesting justification for requests or demands regarding junk food restrictions, chores, proper etiquette or hygiene. One mother described an incident in which she had to explain proper etiquette. “He will challenge you. Ask you questions why, so you have to explain and once you explain everything is fine” (LC, 10-year-old son). Non-cooperative negotiations were described as coercive or illegitimate, because they involved nagging, complaining, and whining to avoid carrying out the task. One child in his quest to consume junk food used this strategy, “He whines and complains” (MC, 9-year-old son).

### Mothers’ Appraisal of Children’s Non-compliance/Resistance

While describing instances of children’s resistance, mothers spontaneously described how they evaluated or felt about children’s displays of agency through resistance. Analyses of these appraisals indicated two themes: non-acceptance of children’s resistance and acceptance of children’s resistance.

#### Non-acceptance

Mothers’ non-acceptance of children’s resistance was evident in the disapproving statements or reports that their children’s non-compliance negatively impacted on parental authority or the parent–child relationship. Most mothers of both social classes reported that they did not accept children’s non-compliance. Many mothers conveyed non-acceptance by describing negative emotions such as feeling angry, upset, annoyed, or disappointed regarding personal safety and etiquette. For example, “The emotion is anger. It makes me feel very angry, angry at him” (MC, 10-year-old son). Some felt upset. “I get very upset with her because appearance means a lot to me; you are coming to my office nothing is supposed to be showing” (MC, 9-year-old daughter). Others reported feeling annoyed when their children talked back, “I was upset because you know I don’t like how she talk to me, how she responded” (LC 12-year-old daughter).

An interesting feature of Jamaican mother’s narratives was that mothers interpreted children’s resistance as having negative implications for their views of themselves as parents. Rather than interpreting resistant behaviors as expressions of child agency, mothers attributed children’s resistance as evidence of their own failure as a parent. One mother described worrying about what she had done wrong in the past. “He also let me think, hmm, was there something that I didn’t do or I didn’t reinforce? Something strong enough for him to falter in that area… Sometime it makes me feel, where I went wrong” (MC, 10-year-old daughter). Another explained how she was emotionally devastated in her identity as parent because of her failure to train her child to be obedient.

Hurt, in the midst of it you’re supposed to be training your child, your different steps, and measures are not working. Things are going to be extreme…I felt hurt to my most, my inner most being, and am trying to raise my son in the way that I think is the best way, and he needs to understand that there are consequences (MC, 10-year-old son).

Some mothers reported that their negative reactions were so intense they had to regulate their emotions and exercise self-control to protect their children from harm or for the sake of maintaining their relationships with their children when they ignored their personal safety request to stay in the home. For example, “Like I say, me get ignorant, (in the colloquial language ignorant means extremely upset), but sometimes I try to control it because I don’t want to hit her…because I don’t like to hit my kids” (LC, 12-year-old daughter).

#### Acceptance of Resistance

A small number of middle class and lower class mothers indicated that they tolerated children’s resistance because they interpreted resistance as a normal expression of autonomy. One mother reported that although her son’s opposition to her request to complete chores was irritating, it was an ordinary part of family life. “You know I expect this in life, so it don’t make me feel no way. Sometimes it can get a little bit on your nerve” (MC, 12-year-old son). Another parent perceived that resistance complete chores was an expected part of adolescent development. “I don’t feel no way; I was saying to myself that because she getting big she feel like seh you don’t understand [that] she is changing. Everything is changing, she is getting big now. She don’t want to do this, she don’t want to do that” (LC, 12-year-old daughter).

Some mothers reported that they were not opposed to their children’s resistance, but they opposed the unskillful way in which their children expressed their opposition. For example, some mothers said they were comfortable when their children resisted verbally by negotiating in a socially appropriate manner. “A think it’s a good thing when she is articulating more as long as the articulation is not disrespectful” (MC, 10-year-old daughter). “I don’t have a problem with it; it always has to be the tone. I always talk to him about the tone, put up your argument, but watch the tone” (MC, 9-year-old son). These mothers appeared to accept their children’s autonomy, but focused on improving their children’s social skills for expressing autonomy appropriately.

### Mothers’ Disciplinary Responses to Children’s Non-compliance and Resistance

Mothers’ reports of their responses to children’s resistance revealed two themes: power assertion and autonomy support. Although mothers from both social classes responded with power assertion that was unilateral and firm, there was also evidence that some mothers responded in a way that allowed give and take in the relationship.

#### Power-Assertion

Power-assertion consisted of strategies by which parents used their greater power in the relationship to compel their children to comply or submit to their rules and requests. Mothers used four power assertive strategies: *physical punishment, psychological control, forced compliance, and deprivation of privileges*.

Fifty percent of parents reported that they used *physical punishment*. Most lower class mothers reported that they used physically coercive consequences for children’s non-compliance and were forthright in their descriptions. For example, one mother said, “A pinch him up” (LC, 10-year-old son). Another said she had her son continuously write lines, until his fingers hurt. “He had was to write discipline lines in a book about hundred lines” (LC, 12-year-old son). Another mother reported slapping her daughter across the face. “Me did have to slap har [her]…” (LC, 12-year-old daughter). Somewhat fewer middle class mothers reported using physical punishment and employed more euphemistic terminology to their approach. These included smacks such as, “I smacked her on her real hard on leg though” (MC, 9-year-old daughter); and spanks, “The last resort of punishment is spanking with a belt which I do occasionally but I try not to do it too much because I know it will cease to have effect” (MC, 11-year-old daughter).

*Psychological control* included mothers’ psychological techniques such as inducing guilt, shame, and threatening to withdraw love. More lower class mothers than middle class mothers (**Table 3**) reported using psychological control. One middle class mother described calling her child’s bluff by feigning indifference to the child’s threats to leave home.

**Table 3:** Themes in mothers’ emotional appraisals and disciplinary responses to children’s non-compliance and resistance.

	Middle	Lower	Total
	(*n* = 17)	(*n* = 13)	
	class	class	(*N* = 30)
**Emotional appraisal**			
Non-acceptance	77% (13)	77% (10)	77% (23)
Acceptance	24% (4)	23% (3)	23% (7)
**Power-assertion**			
Physical punishment	35% (6)	69% (9)	50% (15)
Psychological control	24% (4)	46% (6)	33% (10)
Forced compliance	53% (9)	77% (10)	63% (19)
Deprivation of privileges	77% (13)	39% (5)	60% (18)
**Autonomy support**			
Reasoning	65% (11)	15% (2)	43% (13)
Accommodation	29% (5)	39% (5)	33% (10)


*Table includes percentage of mothers. Absolute values are in parentheses.*

He tried to get us by saying he was going to move out. We told him that we would help him pack; so, we say, “Where you going to go”? “Do you intend to come back”? “What’s the plan”? And so, he is shocked. He is sitting there and say, ‘These people are trying to get me, am going to find another way’ so he just sits there trying to think of another way (MC, 10-year-old son).

Some mothers also explained that they used withdrawal of love despite being aware of the effects of threatening the child’s sense of security in the relationship. “I watch to see how else he is doing and how he is taking it and depending on how he is behaving in his calling I will strategize and for about a week I didn’t talk to him and he said, ‘Daddy, mommy not talking to me”’ (MC, 10-year-old son). Shaming was the only strategy reported by lower class mothers. “Mi call har couple names deh weh nuh lovely” (LC, 12-year-old daughter).

*Forced compliance* refers to mothers’ use of physical force or verbal threats of force in response to children’s attempts to resist a request. Although more than half of the mothers reported using force, lower class mothers appeared to be more intense in this strategy than middle class mothers. One mother described how she physically compelled her child to comply with hygiene rules: “You have to carry him all in d bachroom.” “Listen, brush yuh teet now. Not leaving until yuh finish” (LC, 10-year-old son). Lower class mothers more frequently threatened physical punishment.

Sometime him like to mumble. As you are getting older you’re supposed to learn how to wash the dishes. Am not giving you any pot to wash, wash the dishes and a hear a little sound, and a say excuse me and he said am sorry. And a said don’t do it again because you will lose you front teeth (LC, 12-year-old son).

Mothers from both classes reported the use of ‘shouts’ to gain compliance. Although some were uncomfortable with resorting to ‘shouts,’ they argued that it was effective. “Sometimes I will just shout. I know I shouldn’t do it, but if a do it once he hears” (MC, 12-year-old son). “She lie dung dere couple minutes pon har phone until I have to shout and tell har seh mi a come in dere fi har suh when she actually get up. It’s frustrating” (LC, 12-year-old daughter).

*Deprivation of privileges* such as enjoyable activities or use of possessions was reported more often by middle class mothers. For example, one mother said: “School was ending at that time so she didn’t go to the school Fair” (MC, 9-year-old daughter). A middle class mother said she used deprivation of privileges as a non-coercive alternative to physical punishment. “I don’t believe in hitting a child so withdrawal of privileges is very effective. When you take away that IPad they miss that so it works” (MC, 11-year-old daughter). However, some lower class mothers also reported using deprivation of privileges as a consequence. “I think it’s some party thing or some school thing and I never send her” (LC, 10-year-old daughter).

#### Autonomy Support

In contrast to power assertion where mothers unilaterally imposed their will on their children, autonomy support consisted of responses that attempted to win over children’s voluntary cooperation through verbal reasoning or persuasion or accommodate the children’s perspective. *Reasoning* involved mothers engaging in dialogue which allowed children to share their perspectives on parental expectations. Eleven middle class and only two lower class mothers used this approach (**Table 3**). “I spoke to her about the situation and she explained, “Mommy he hit me first” he started it and she was just reacting. We spoke about the situation and we both came to a resolution” (MC, 11-year-old daughter). Some mothers reported that they used reasoning to teach children the importance of regulating their emotional reactions as a form of respect for others. “I said, you can’t be like that. I understand that you are upset and disappointed that we are here earlier and it is less time at the nursery to play games etc., but then we are adults you can’t be speaking to us like that” (MC, 8-year-old daughter). Reasoning was also used to explain the consequences of actions involving safety and protection. One mother described her ‘matter of fact’ approach in explaining the dangers of unregulated internet surfing:

Dangers of surfing the internet. I figured what I needed to do was to have a discussion with him about why it’s not a good idea because I was explaining to him, you are using my account to search for things and when using my account to search for things the internet is not a private place and people do track you through the internet. They observed what you watching (MC, 9-year-old son).

*Accommodation* was less reported and consisted of mothers relaxing or modifying their expectations of their children’s responses to requests/demands to fit their children’s needs. In these interactions mothers allowed their children to negotiate the terms of fulfilling the requests/demands. Some mothers did not see the need to be overly strict with their children. For example, one mother said, “I break the rules for her also. In the case where I feel I don’t have a lot of time because of work, so I will break the rules for her” (LC, 9-year-old daughter). One mother explained that while it was important to have manners, it was not something that had to be rigidly adhered to. “I think it is important to apologize but, sometimes I think it is okay. It’s kind of like saying please and thank you. You don’t have to do it a 100% of the time” (MC, 11-year-old daughter).

## Discussion

Previous research found that Jamaican parents use an authoritarian parenting style emphasizing obedience and harsh disciplinary consequences. The findings of this study are consistent with this characterization but contribute a more detailed understanding of what these parent–child interactions entail. Using the theoretical premise that socialization is a bidirectional process to which parents and children contribute (Kuczynski, 2003), this study not only described Jamaican mothers’ interpretations and actions as agents in children’s socialization. It also identified in mother’s narratives an implicit recognition that their children act as agents who attempt to achieve their own goals when they are blocked and defend their autonomy despite their mothers’ sometimes harsh efforts to control them.

The findings on children’s resistance strategies are novel to the Jamaican literature. Mothers reported that their children evaded or challenged mothers’ authority both directly and assertively, and indirectly and subversively. This range of resistance strategies, reported were like those found in middle class North American studies (Kuczynski and Kochanska, 1990; Parkin and Kuczynski, 2012; Kuczynski et al., 2013). As in previous research, Jamaican mothers’ descriptions of resistance strategies can be understood as manifesting variations in assertiveness and social skill (Kuczynski and Hildebrandt, 1997). Non-confrontational strategies such as ignoring can be interpreted as an unassertive strategy because children attempted to evade complying without directly communicating their opposition to parents. In contrast, self-assertion, arguing, negotiation are assertive strategies because children directly confronted parents with their opposition. The other dimension is social skill.

Children’s resistance strategies can be interpreted as attempts to persuade parents to modify their demands. In this regard, strategies such as simple assertion and negotiation are more skillful and less likely to arouse resistance from parents than resisting by ignoring or arguing (Dix et al., 2007; Grunzeweig et al., 2009; Parkin and Kuczynski, 2012). Negotiation, the most sophisticated form of resistance has been previously reported throughout childhood in Western cultures (Parkin and Kuczynski, 2012), African American (Smetana et al., 2003) and Asian families (Chen-Gaddini, 2012; Goh and Hsu, 2013). Negotiation involves children’s attempts to engage a bidirectional process of give-and-take with their mothers with the goal of accommodating parental wishes but still meeting their own goals.

Also, consistent with North American studies, Jamaican mothers reported that their children challenged their authority by displaying attitude. Attitude is a subversive often non-verbal expression of agency by which children who have been compelled to comply behaviorally, communicated their disapproval of the parent’s expectation (Kuczynski et al., in press). Although many of the displays of attitude reported for Jamaican children such as eye rolling and dismissive vocalizations are similar to those reported in North American studies, mothers also said, that their children communicated resistance by “hiss-teeth,” a unique form of dismissive expression that may be specific to Afro-Caribbean culture. Hiss-teeth was the only form of attitude that was reported by lower class mothers. The action is a gesture of disrespect and communicates emotions of annoyance, displeasure, resentment, scorn, impatience or disdain (Figueroa and Patrick, 2002). Hiss-teeth may be an indirect and safer way of signaling that they do not accept the imposition of parental messages and that their cooperation is obtained under duress (Scott, 1990; Rubenstein and Feldman, 1993).

An important aspect of these resistance strategies is that although Jamaican parents emphasize strict rules, demand obedience, provide severe consequences for non-compliance (Crawford-Brown, 1999), they nevertheless reported that their children regularly resisted their parental attempts to control their behavior. Although a traditional unilateral perspective on socialization (McMahon and Forehand, 2003) might interpret this as a failure of parental discipline, the same findings can be interpreted using the developmental lens of resistance and suggest that regardless of the extremity of parental control, children will find ways to express their agency. This is also consistent with previous studies that identify children’ motives for autonomy in other collectivistic cultures (Smetana, 2002; Helwig et al., 2003).

The Jamaican mothers in this study overwhelmingly expressed their disapproval of their children’s resistance, which is consistent with previous findings of the authoritarian nature of Jamaican parents (Smith and Mosby, 2003). However, this finding can be contrasted with North American studies which found that middle class mothers tended to tolerate resistance as a normative and acceptable expression of their children’s developing autonomy (Kuczynski and Hildebrandt, 1997; Kuczynski et al., 2013).

The findings contribute to the development of the cross-cultural literature by highlighting the co-existence of high resistance. As a collectivistic culture, it is expected that there would be harmony, communalism, obedience and great interdependence in the mother-child relationship, which would minimize children’s resistance. However, this study presents a more complex and multi-dimensional picture of relational dynamics. Specifically, mothers described children as being just as concerned with maintaining their autonomy, as in cultures that score high in individualism, rather than primarily preserving expected harmony or obedience. The explicit presence of resistance is consistent with and confirms Powell’s (2009) findings of the equal coexistence of collectivistic and individualistic goals.

Mothers predominantly responded to children’s resistance with coercive forms of discipline such as corporal punishment and psychological control. A qualitative feature of mothers’ narratives was the transparently harsh terminology they used to describe their own actions. Although middle class mothers appeared to use euphemistic terms such as “spanking” instead of “beating” for corporal punishment than lower class parents, it seemed that Jamaican mothers were not fettered by concerns about social desirability and indeed appeared to convey a sense of moral righteousness in their approach to discipline. Lower class mothers more often reported using physical punishment, psychological control, and force whereas middle class mothers more often reported using reasoning and withdrawal of privileges.

Although the qualitative nature of the study prevents making inferences, it is noted that potential numerical differences in the kinds of disciplinary strategies reported by the different social classes are consistent with findings of previous research. The use of physical punishment, especially among lower class families, is consistent with earlier findings among the population (Brown and Johnson, 2008; Ricketts and Anderson, 2009; Smith et al., 2011; Smith and Moore, 2013). Moreover, lower class parents more often reported psychologically controlling tactics such as inducing guilt, shame, fear, and insecurity by the deliberate withdrawal of parental love (Barber and Harmon, 2002). These findings are also consistent with studies elsewhere that found that lower social class families are more likely to use psychological control to reduce misbehavior (Hoff-Ginsberg and Tardif, 1995; Chen et al., 2015). The use of mostly power assertive strategies among lower class families may be attributed to a lack of knowledge to alternative options (Burke and Sutherland, 2014), or they may experience more economic challenges that reduce their patience level compared to middle class families.

The findings suggest that positive parenting practices are also present within Jamaican families, with middle class families apparently taking the lead. These positive practices included non-violent forms of discipline such as withdrawal of privileges (McMahon and Forehand, 2003). They also include autonomy supportive practices of providing cognitive explanations (Ricketts and Anderson, 2009) and accommodating children’s perspectives when deemed appropriate. Autonomy-supportive strategies support children’s experience and expression of agency by acknowledging children’s perspectives and desires and providing meaningful rationales for decisions (Grolnick et al., 1997). This parental decision ensured that cooperation is self-accepted rather than externally compelled by fear of punishment. The use of these positive practices may be a result of the gradual Americanization of Jamaican parental behavior by exposure to popular media (Ferguson and Iturbide, 2015). On a relational level, it implies that although Jamaican mothers are described as authoritarian, many appear to be willing to balance the power dynamics by considering children’s perspective through concession. The findings suggest that the definition of authoritarian parents, which was created using western families may not apply to ethnic Afro-Caribbean groups.

The few studies that have considered children’s behaviors and actions suggest that Jamaican children actively contribute to the nature of parent–child relationships. For example, Burke et al. (2017) found that parents perceived that their children contributed constructively to parent–child relationships by maintaining closeness and intimacy with their mothers despite harsh discipline practices. There is also evidence that Jamaican children do not passively accept the kinds of parenting that they receive. Researchers have found that Jamaican children disapproved of their parents’ use of punitive parental control strategies and were perplexed by their parents’ justification that such practices were an expression of their love (Bailey et al., 1998; Brown and Johnson, 2008).

The findings of the study have clinical implications which warrant future research. Intervention programs and research may need to focus on the relational aspect of the relationship when addressing children’s resistance to parental control. This shift will entail a move away from behavior management perspectives that emphasize immediate compliance without regard to cognitive processes that allow co-regulation and co-interpretation of the interactions in children (Cavell, 2001). Concerning mothers’ emotional and behavioral responses to resistance, parents need to be educated that children’s resistance is normative and demonstrates the development of autonomy, which is needed to enhance a sense of efficacy and independence (Grolnick, 2003). The dissemination of this knowledge may change the focus of Jamaican parents away from suppressing resistance to guiding children’s development of skills to express themselves in a socially competent manner. Van Petegem et al. (2015) found that only unskillful oppositional forms of non-compliance are associated with negative outcomes for children.

Continued education on the negative effects of harsh power-assertive strategies on children’s well-being and the health of the mother–child relationship is necessary (Gershoff et al., 2012). However, based on the present findings we would argue that many Jamaican mothers may have unrealistic expectations of children’s strict obedience as a goal, as well as their own capacity to determine this by coercive means. A telling finding is that some mothers interpreted their children’s resistance as their own failure in parenting, which had negative emotional consequences for mothers themselves. This suggests mothers may have a linear or deterministic perspective on their own influence, which implies that they are the sole cause of children’s outcomes.

Kuczynski and De Mol (2015) argued that successful parenting requires a relational as opposed to a deterministic perspective on interpersonal influence. A full recognition that children are agents who exert their influence on outcomes requires an agent–agent perspective which accepts that linear control is impossible and that children as agents will have their input in interpreting, resisting or inserting novelty into social interactions and socialization outcomes. Morrissey and Gondoli (2012) found that mothers with young adolescents who engaged in assertive but well-regulated non-compliance tended to have positive perceptions of their influence on their children’s behavior.

This study had limitations that may have impacted the findings. First, the study only focused on mothers’ perception of children’s resistance strategies, which resulted in an incomplete picture of contexts of the interactions. The inclusion of children’s perspectives would have resulted in richer details and understanding of relational dynamics. Second, mothers were asked to randomly choose one of their children to be the subject of the interview. There is a possibility that mothers chose the child who they had the best relationship. Third, we did not examine the difference in resistance strategies based on children’s gender. There may be a possibility that boys resist differently from girls. Fourth, we did not investigate whether there were differences in the way that mothers from the social classes imposed rules and requests. There is a possibility that children’s reactions were based on the mothers’ demeanor during the interaction. Fathers’ perspectives were also not included, so it is difficult to determine whether they have similar emotional and behavioral responses to children’s resistance. Finally, the qualitative nature of the findings, particularly regarding social class, require replication with larger quantitative studies that permit inferences of statistical difference.

## Conclusion

This study indicates that despite strong cultural values for obedience and submission to authority both middle class and lower class Jamaican mothers nevertheless perceive children as exercising their agency by means of resisting attempts to control their behavior. Although there were some differences in cultural expression of resistance, Jamaican children’s repertories of resistance strategies resembled those found in other cultural samples indicating that children’s use of resistance as an expression of agency may be culturally universal. Furthermore, unlike mothers from individualistic cultures, Jamaican mothers were uncomfortable with resistance and used a combination of punitive and non-punitive measures to suppress it.

## Ethics Statement

The University of Guelph Research Ethics Board as well as the University of the West Indies (Mona) Ethics Committee granted ethical approval. Participants signed consent forms before the commencement of the interviews. They were asked to participate in an open-ended 1 to 1.5-h semi-structured interview in their home or a mutually convenient location.

## Author Contributions

TB conceptualized, planned, and conducted the research project and also analyzed the data and wrote the paper. LK supervised the execution of the project, independently reviewed the results, and revised the draft versions of the paper. Both authors read and approved the final manuscript.

## Conflict of Interest Statement

The authors declare that the research was conducted in the absence of any commercial or financial relationships that could be construed as a potential conflict of interest.
